# Modeling pluralism and self-regulation explains the emergence of cooperation in networked societies

**DOI:** 10.1038/s41598-021-98524-5

**Published:** 2021-09-28

**Authors:** Dario Madeo, Sergio Salvatore, Terri Mannarini, Chiara Mocenni

**Affiliations:** 1 Department of Information Engineering and Mathematics, University of Siena, Via Roma, 56, 53100 Siena, Italy; 2 Department of Dynamic, Clinic and Health Studies, Sapienza University of Rome, Via degli Apuli, 1, 00183 Rome, Italy; 3 Department of History, Society and Human Studies, University of Salento, Via di Valesio, 73100 Lecce, Italy

**Keywords:** Applied mathematics, Human behaviour

## Abstract

Understanding the dynamics of cooperative behavior of individuals in complex societies represents a fundamental research question which puzzles scientists working in heterogeneous fields. Many studies have been developed using the unitary agent assumption, which embeds the idea that when making decisions, individuals share the same socio-cultural parameters. In this paper, we propose the ECHO-EGN model, based on Evolutionary Game Theory, which relaxes this strong assumption by considering the heterogeneity of three fundamental socio-cultural aspects ruling the behavior of groups of people: the propensity to be more cooperative with members of the same group (Endogamic cooperation), the propensity to cooperate with the public domain (Civicness) and the propensity to prefer connections with members of the same group (Homophily). The ECHO-EGN model is shown to have high performance in describing real world behavior of interacting individuals living in complex environments. Extensive numerical experiments allowing the comparison of real data and model simulations confirmed that the introduction of the above mechanisms enhances the realism in the modelling of cooperation dynamics. Additionally, theoretical findings allow us to conclude that endogamic cooperation may limit significantly the emergence of cooperation.

## Introduction

The modelling of the evolution of cooperation in social networks is a consolidated stream of research at the boundary of mathematics and social sciences. The main focus of this line of work is the understanding of how cooperation can develop in a population of agents based on selfish motivations. Evolutionary Game Theory assumed the role of main framework (e.g.^1^), with many studies adopting the Prisoner Dilemma as analytical tool to model the emergence of mutually beneficial interactions among decision makers^2–7^.

These studies model the population dynamics under the basic assumption of the unitary agent, namely the idea that the social network is comprised by agents following the same model of decision making, based on the optimization of own utility. This assumption finds expression in two main computational settings—first, the invariance of the decision-maker—i.e. all agents follow the same model of choice, for instance that based on the maximization of utility; second, the well-mixed population design—i.e. the fact that the agent has generally the same chance of interaction with all others.

The first aspect has been relaxed in several studies, where individuals with heterogeneous decision making rules—for example, that players use different types of payoff matrices^8,9^, or heterogeneous decision-making functions^10,11^—have been considered. Moreover, it has been shown that cooperation is fostered when imitative and innovate decision-making rules^12^ coexist.

Regarding the second aspect, it has to be noted that in recent years more complex models of population have been introduced, characterized by spatial structures, like lattices^13^. Additionally, more complex organized structures have been taken into account, assuming that agent interactions take place according to the topology of a network of inter-connections (e.g.^6,7,14^). Remarkably, the degree distribution of the underlying network is able to affect the game dynamics^14–16^.

These developments provided a contribution to overcome the idealized approach implied in the unitary agent assumption that limits the realism of research and therefore the chance to model natural social settings for the sake of understanding the current and future actual evolution of historically concrete human groups, which are inherently plural (e.g.^17–19^). The latter aim requires an output-centered approach, namely an approach which is mainly interested in understanding the potential evolution of given input states, and to draw from it the identification of structural and individual conditions improving the overall level of cooperation.

The aim of this paper is to present a contribution in that direction, by proposing an evolutionary game model of cooperation that takes into account the self-regulation characteristics of the actors and the cultural pluralism of societies. The model, called ECHO-EGN, integrates the psycho-social theory of the inherent pluralism of social networks in the framework of Evolutionary Game Theory. ECHO-EGN is an output-centered model, which is expected to have both theoretical and application values. From a theoretical perspective, it increases the ecological validity of current models, and in so doing it makes the formal analysis of cooperative dynamics more complete. From an application standpoint, it enables efficacious simulations of the evolution of cooperative scenarios for the sake of policy decision making.

The purpose of the paper is twofold. First, it intends to present and to validate ECHO-EGN; second, based on this first result, it analyses the model in order to highlight its capacity to identify relevant conceptual properties of the evolution of cooperation in social networks.

The paper is organized in the following way. First, we discuss critically the unitary agent assumption in the light of the psycho-social conceptualization of the relation between mind, culture and society. Second, the ECHO-EGN model is presented, proposed as an extension of a previous model (EGN) aimed at accounting for the presence of different groups of agents. Third, an empirical validation of the model is provided, based on a simulation design—the observed levels of cooperation in a cluster of actual social networks is compared with the levels obtained by corresponding ECHO-EGN simulations. Fourth, analytic components of the model are developed in order to shed light on the properties of cooperative dynamics. Discussion and conclusions are devoted to highlighting the elements of interest of ECHO-EGN as well as limitations and perspectives of the current stage of its development.

## The cultural variability of social networks

The last three-four decades have witnessed the progressive rediscovering of the role culture plays in political and economic affairs by all social sciences (e.g.^20–24^). At the boundaries between psychology, economics, political science and sociology, the concept of social capital^25,26^ has provided a view of social behavior, and more in general of the functioning of society and institutions, as depending on the incidence of trust—namely, a factor deeply rooted in cultural contexts.

Within this general perspective, several authors have proposed a view of culture as the *source of human variability*. According to this view, culture is a network of complementary and conflicting meanings (e.g.^17,24^); members of a population share the same network of meanings, yet they assume different positions in it—i.e. they adhere to a certain subset of meanings (e.g. a given worldview^27^, a system of values^23^), thus rejecting the conflicting ones. As result, each subset of meanings frames the way of thinking and acting of the segment of the population adhering to it. In doing so, the shared network of meanings works as both the basic common ground and what makes members of the social group different from each other.

ECHO-EGN focuses on three major sources of cultural pluralism of the social group (for a recent analysis of the role these three factors play in the cultural differentiation of a set of European societies, see^28,29^).

### Homophily

Homophily is the propensity of an individual to prefer connections with members of the same group instead of the out-groups. For instance, McPherson and colleagues(^30^, p. 416) define it as the tendency for friendships “between similar people [to occur] at a higher rate than among dis-similar people”. As it was highlighted (e.g.^31,32^) Homophily is distributed heterogeneously within the social group, as a result of cultural drivers.

### Endogamic cooperation

Here we use this concept in the broad sense, to denote the propensity to be more cooperative with members of one’s group than with members of other groups. Cultural segments differ as to the degree of endogamic cooperation. Familist cultures^33^ as well as cultures fostering identity motives^34^ tend to increase the member’s endogamic cooperation, namely to make them more inclined to cooperate with in-group members than with out-group members. In contrast, universalist values make adherents cooperate with in-group and out-group similarly^35^.

### Civicness

Any social interaction is embedded in a web of institutions—formal and informal norms and underpinning meanings that make individual actions interconnected. As used here, Civicness consists of the valorisation of such embeddedness^34,36^. Accordingly, Civicness can be conceived of as the extension of the propensity to cooperate to the public domain—namely, to the relation with what is extraneous (^17^, chapter 9): it consists of the actors’ capacity of self-regulation, by reason of the rules of the collective game underlying the production of common goods^37^.

## Evolutionary games for culturally plural social groups: the ECHO-EGN Model

The ECHO-EGN Model is a development of a previous model. In its first version—the Evolutionary Game on Network equation (EGN), it was designed to account for specific characteristics of individuals, beyond the assumption of the unitary agent. EGN described the dynamical evolution of the cooperation of each player, located inside a network of connections, which is engaged in several 2-player games with neighbors over time. Thus, EGN introduced a variable distribution of connections in the mathematical modelling of social networks (cf.^38,39^). A further element able to foster the presence of differences among individuals was introduced in subsequent papers, where self-regulation mechanisms were considered in the framework of the prisoner’s dilemma^40^. Specifically, the self-regulation mechanisms act as fundamental drivers able to promote cooperation at the local and global levels^41^.

The extended version of the original model (called SR-EGN)^41^ considers a population of *N* individuals, $v \in \{1, \ldots , N\} = \mathcal {V}$ arranged on an undirected graph of connections, defined by the symmetric adjacency matrix $\mathbf{A} = \{a_{v,w}\} \in \{0,1\}^{N \times N}$. When $a_{v,w} = 1$, then *v* and *w* are neighbors, while $a_{v,w} = 0$ means that *v* and *w* are not connected. We will refer to the number of neighbors of a generic player *v* as its degree, then $k_v = \sum _{v=1}^N a_{v,w}.$

The topology of the connection network among individuals is assumed to be random with a scale-free distribution and average degree $\overline{k}$^3,42,43^. Of note, the random distribution makes agents differ as to their connectivity; this is consistent with the assumption that connectivity is distributed heterogeneously over the social group, as a result of cultural norms—(e.g.^31,32^). Moreover, assumption of a power-law distribution of connections (scale-free network) is grounded on well-established findings on real world communities^42^. This fact has been also confirmed in other studies, when heterogeneous groups are present within the social network^46^.

Each member of the population plays 2-player games with all its neighbors continuously over time. The games played are assumed to be Prisoner’s dilemmas, where the payoff earned by player *v* against *w* is described by the matrix:

$$\begin{aligned} \mathbf{B}_{v,w} = \begin{bmatrix} R_{v,w} &{} S_{v,w} \\ T_{v,w} &{} P_{v,w} \end{bmatrix}, \end{aligned}$$

where $R_{v,w}$ is the reward for mutual cooperation, $T_{v,w}$ is the temptation to defect when the opponent cooperates, $S_{v,w}$ is the sucker’s payoff earned by a cooperative player when the opponent is a free rider, and $P_{v,w}$ is the punishment for mutual defection. A Prisoner’s dilemma game is characterized by the relation $T_{v,w}> R_{v,w}> P_{v,w} > S_{v,w}.$ In this work, we assume that $R_{v,w} = 1$, $P_{v,w}=0$, $T_{v,w} > 1$ and $S_{v,w} < 0$. Moreover, we assume that the temptation to defect is stronger than the fear of being betrayed, i.e. $T_{v,w}-1 > -S_{v,w}$.

According to^41^, each player plays also a game against itself, which acts as a self-regulatory term. Indeed, it is known that in human societies and animal groups, self-mechanisms are recognized able to contrast selfish behaviors, thus making possible the pursuit of cooperation resulting from personal awareness and culture^44,45^. Using self-games is a simple way to embed into the mathematical model internal evaluations, such as “what kind of reward would I earn if I use a given strategy against myself?”. We denote with $\mathbf{B}_{v,v}$ the payoff matrix related to this game, and with $\beta _v$ the strength of the self game. Notice that the self-game can be different with respect to the standard game^41^.

Thus, taken as a whole, the level of cooperation of a generic player *v* is denoted by $x_v \in [0,1]$, and its dynamics is ruled by the following equation:

1 $$\begin{aligned} \begin{array}{rcl} \dot{x}_v &{} = x_v(1-x_v) &{} \Bigg \{{\displaystyle \sum _{w = 1}^N a_{v,w}} \left[ (1-T_{v,w}-S_{v,w}){x_w} + S_{v,w}\right] \\ &{}&{} -\beta _v\left[ (1-T_{v,v}-S_{v,v})x_v + S_{v,v}\right] \Bigg \}, \end{array} \end{aligned}$$

where $\dot{x}_v$ denotes the time derivative of $x_v$, i.e. $\dot{x}_v = \mathrm {d}x_v/\mathrm {d}t$.

In a nutshell, this equation states that the steady state level of cooperation is one among full defection, full cooperation and intermediate values of cooperation/defection. The effective level of cooperation is then reached according to a selection mechanism ensuring the maximization of the population reward, together with the satisfaction of self-regulation mechanisms depending on parameter $\beta _v$. Thus, the latter acts as an inertial factor able to counteract the natural tendency of individuals towards defection.

### The ECHO-EGN group-specific features of cultural variability

In order to represent the cultural variability of the social network—i.e. the culturally driven heterogeneous distributions of Homophily, Endogamic cooperation, and Civicness among agents—the ECHO-EGN model adopts a grouped population design.

The population $\mathcal {V}$ is assumed to be subdivided into *M* groups, namely $\mathcal {G}_{1}, \ldots , \mathcal {G}_{M}$, such that $\bigcup _{g=1}^M \mathcal {G}_{g} = \mathcal {V},$ and $\mathcal {G}_{g} \cap \mathcal {G}_{j} = \emptyset$, for all $g \ne j.$ The size of group $\mathcal {G}_{g}$ is $N_g$. Hence, the share of population belonging to group $\mathcal {G}_{g}$ is $\delta _g = \frac{N_g}{N} \in (0, 1)$. Each group is assumed to have a scale-free distribution of the degrees, with average equal to $\overline{k}_g$.

When playing a game, the individual distinguishes between members of the same (*affine*) and of different (*non affine*) groups. From now on, the corresponding quantities will be indicated by the superscript $\mathrm {A}$ for affine players, and $\mathrm {N}$ for non affine players. For example, $k^{\mathrm {A}}_v$ and $k^{\mathrm {N}}_v$ are the number of links of player *v* with affine and non-affine players, respectively, i.e. $k^{\mathrm {A}}_v = \sum _{w \in \mathcal {G}_g} a_{v,w}$ and $k^{\mathrm {N}}_v = \sum _{w \in \mathcal {V} \setminus \mathcal {G}_g} a_{v,w}.$ Notice that $k_v = k^{\mathrm {A}}_v + k^{\mathrm {N}}_v$.

In the following subsections, the ECHO-EGN model specifications of Homophily, Endogamic cooperation, and Civicness will be introduced.

#### Homophily

As stated above, Homophily is the propensity of an individual to prefer connections with members of the same group. In order to account for this property, a rewiring process has been carried out to modify the initial network of connections, according to a given probability, specific for each group, and denoted by the Homophily factor $h_{g} \in [0, 1]$. All details on the algorithm used for the rewiring phase are given in Appendix A.

#### Endogamic cooperation

As stated above, Endogamic cooperation is the tendency of individuals to be more cooperative with members of the same group (affine players). The parameter $e_g$ affects the structure of the payoff matrix played in games with affine individuals. Specifically, given $v \in \mathcal {G}_g$ and $w \in \mathcal {V}$ with $a_{v,w} = 1$, we define:

$$\begin{aligned} \mathbf{B}_{v,w} = {\left\{ \begin{array}{ll} \mathbf{B}^{\mathrm {N}}&{} \text {if}~ w \not \in \mathcal {G}_g \\ \mathbf{B}^{\mathrm {A}}_g &{} \text {if}~ w \in \mathcal {G}_g \end{array},\right.} \end{aligned}$$

where

$$\begin{aligned} \mathbf{B}^{\mathrm {N}}= \begin{bmatrix}1 &{} S^{\mathrm {N}}\\ T^{\mathrm {N}}&{} 0\end{bmatrix} \end{aligned}$$

is the payoff matrix used with non affine players, and

$$\begin{aligned} \mathbf{B}^{\mathrm {A}}_g = \begin{bmatrix}1 &{} S^{\mathrm {A}}_g\\ T^{\mathrm {A}}_g &{} 0\end{bmatrix}, \end{aligned}$$

is the payoff matrix used with affine players, where

2 $$\begin{aligned} T^{\mathrm {A}}_g = (1-e_g)T^{\mathrm {N}}\end{aligned}$$

and

3 $$\begin{aligned} S^{\mathrm {A}}_g = (1-e_g)S^{\mathrm {N}}. \end{aligned}$$

Coherently, the payoff matrix of the self-game is $\mathbf{B}_{v,v} = \mathbf{B}^{\mathrm {A}}_{g}$.

#### Civicness

An important assumption of the present model is that different groups show different levels of Civicness, named $c_g$. In the SR-EGN model, Civicness is naturally embodied by parameter $\beta _v$, which maps the self-regulation mechanism constraining the agent’s selfish attitude.

Moreover, we assume that all members of a given group share the same self-regulation parameter, which depends on the Civicness value $c_g$ of the group, according to the following formula:

4 $$\begin{aligned} \beta _{v} = \rho ^{\mathrm {N}}\overline{k}'_g \displaystyle \frac{(1 + c_g)^2}{2} ~\forall v \in \mathcal {G}_g. \end{aligned}$$

where $\rho ^{\mathrm {N}}= \frac{1-T^{\mathrm {N}}}{S^{\mathrm {N}}}$ and $\overline{k}'_g$ is the effective average degree of group *g*.

**Figure 1:**
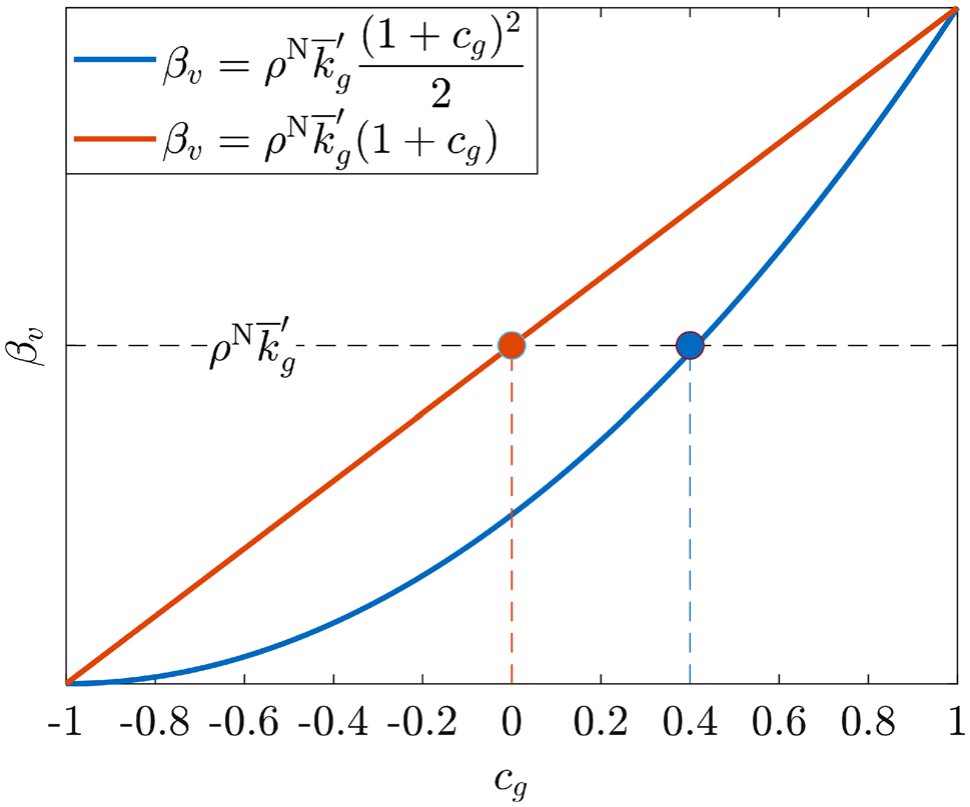
Pictorial representation of the function $\beta _v$ reported in (4) (blue) in comparison with a linear scaling (red). $\beta _v$ exceeds the critical value $\rho ^{\mathrm {N}}\overline{k}'_g$ for $c_g > 0$ in the linear case (red dot), and for $c_g>0.4$ in the quadratic case (blue dot).

This equation assumes that the Civicness parameters $c_g$ determine an increase (when $c_g > 0.4$), a reduction (when $c_g < 0.4$) or no change (when $c_g = 0.4$) in the self-regulation parameters $\beta _v$ with respect to a critical value $\rho ^{\mathrm {N}}\overline{k}'_g$, introduced by Theorem 5 of^41^. Recalling Theorem 5 of^41^, and assuming that temptation is stronger than the fear being betrayed ($T^{\mathrm {N}}-1 > -S^{\mathrm {N}}$), the quantity $\rho ^{\mathrm {N}}\overline{k}'$, where $\rho ^{\mathrm {N}}= \frac{1-T^{\mathrm {N}}}{S^{\mathrm {N}}} > 1$, represents the average threshold for ensuring the global asymptotic stability of the equilibrium $\mathbf{x }^{\text {ALLC}} =[1, \ldots , 1]^\top$ (i.e., the steady state where all individuals fully cooperate).

Based on this result, in the ECHO-EGN model with groups, we assume that the self-regulation parameter of a player *v* in group *g* depends on the average threshold $\rho ^{\mathrm {N}}\overline{k}'_g$ and on the Civicness value $c_g$ as reported in equation (4).

The meaning of the self-regulation parameters $\beta _v$ is discussed in section “Analytical results”. The choice of a quadratic function, reported in blue in Fig. 1, is reasonable since $\beta _v$ exceeds the critical value $\rho ^{\mathrm {N}}\overline{k}'_g$ for higher values of the Civicness parameter with respect to a linear scaling factor $\rho ^{\mathrm {N}}\overline{k}'_g (1+c_g)$ (see red line in Fig. 1). In this way, since values of $\beta _v$ lower than the critical value do not guarantee the convergence of the highest level of cooperation (i.e. $x_v = 1$), using the quadratic function (4) individuals are more free to choose their orientation towards cooperation.

Thus, the label “ECHO-EGN” can be intended both to mean the model’s main purpose—the increase of ecological validity—and the reference to the three parameters of cultural variability—Endogamic cooperation, Civicness, and HOmophily.

### The ECHO-EGN equation

Using these assumptions, Eq. (1) for player $v \in \mathcal {G}_g$ can be rewritten as follows:

5 $$\begin{aligned} \begin{array}{rcccl} \dot{x}_{v} &{} = &{} x_{v}(1 - x_{v}) &{} \displaystyle \Bigg \{ &{} k^{\mathrm {A}}_v\left[ \left( 1-T^{\mathrm {A}}_{g}-S^{\mathrm {A}}_{g}\right) \overline{x}^{\mathrm {A}}_v + S^{\mathrm {A}}_g\right] \\ &{} &{} &{} + &{}k^{\mathrm {N}}_v\left[ \left( 1-T^{\mathrm {N}}-S^{\mathrm {N}}\right) \overline{x}^{\mathrm {N}}_v + S^{\mathrm {N}}\right] \\ &{} &{} &{} - &{}\beta _v\left[ \left( 1-T^{\mathrm {A}}_{g}-S^{\mathrm {A}}_{g}\right) x_v + S^{\mathrm {A}}_{g}\right] \Bigg \} \end{array}, \end{aligned}$$

where

$$\begin{aligned} \overline{x}^{\mathrm {A}}_v = \frac{1}{k^{\mathrm {A}}_v}\sum _{w \in \mathcal {G}_{g}} a_{v,w}x_w \end{aligned}$$

is the affine equivalent opponent of *v*, and

$$\begin{aligned} \overline{x}^{\mathrm {N}}_v = \frac{1}{k^{\mathrm {N}}_v}\sum _{w \in \mathcal {V}\setminus \mathcal {G}_{g}} a_{v,w}x_w \end{aligned}$$

is the non affine equivalent opponent of *v*. More details are available in Appendix B.

Notice that equation (5) is similar to equation (1), provided that the Endogamic cooperation, Homophily and Civicness parameters have been embedded, as described above.

## Model setup and validation

In this section we present the empirical analyses developed for testing ECHO-EGN’s ecological validity, namely its capacity to represent natural social networks. To this end, we adopted a simulation design—we set a cluster of ECHO-EGN models up, in order to make each of them simulate a natural social network corresponding to a European regional area; then, we compare the level and distribution of cooperation of the actual social networks with those of the corresponding ECHO-EGN simulations. Moreover, in order to test that the efficacy of the ECHO-EGN simulation was due to the parameters embedding the cultural variability (i.e. Endogamic cooperation, Civicness and Homophily), the performance of the ECHO-EGN simulations was compared with a control condition—namely, a corresponding simulation carried out by a model approximating the unitary agent assumption. In what follows details, of the method and findings are reported.

### The set-up of ECHO-EGN

The first step for setting up the parameters of the ECHO-EGN equation consists of the generation of the network of connections among agents. The network is generated in two steps. Initially, for each group *g*, a scale-free random network of size $N_g$ and average degree $\overline{k}_g$ is generated. Secondly, a rewiring process is implemented for embedding the Homophily property. Further details of this procedure are reported in Appendix A.

In order to avoid any a priori bias in the group connectivity other than the diversity due to the natural degree distribution of scale-free networks, we assume that all groups share the same average degree, specifically $\overline{k}_g = \overline{k} = 4$. This can be done without loss of generality using the theoretical findings of^41^, for which the thresholds for full cooperation are scalable with respect to the average degree $\overline{k}$.

We assume that individuals play Prisoner’s dilemma games. In particular, the base payoff parameters have been set to $T^{\mathrm {N}}= 4$ and $S^{\mathrm {N}}= -1$. The values of $T^{\mathrm {A}}_g$ and $S^{\mathrm {A}}_g$ for each group, incorporating the Endogamic cooperation parameters, are reported in Table 3.

#### The set-up of cultural parameters

The three cultural parameters (Homophily, Endogamic cooperation, and Civicness) of the ECHO-EGN models were estimated in accordance to the recent cultural map of European societies carried out by Salvatore and colleagues^28^. They surveyed national representative samples of a set of European countries.The investigation led to the identification of five basic worldviews—defined “symbolic universes”—each of them characterizing a cultural group of the population. Moreover, the study provided the distribution of the symbolic universes in each NUTS2 region (see Table 1). The distribution is given in terms of the size of the segments of population, each of them defined by individuals characterized by one of five symbolic universes described below.

1. *Ordered universe group* ($\mathcal {G}_1$). The world is a nice place to live; The positive view concerns every aspect: institutions, services, future, perceived as trustworthy. Endorsement of transcendent values (e.g., justice, solidarity).
2. *Interpersonal bond group* ($\mathcal {G}_2$). Interpersonal bonds and the emotional experience of being involved in them is what matters in life.
3. *Caring society group* ($\mathcal {G}_3$). Institutions are responsive to individual needs. They support people in accomplishing their projects.
4. *Niche of belongingness group* ($\mathcal {G}_4$). The world is a threatening place. The primary network is the shelter from it. Belongingness is the way to survive.
5. *Others’ world* ($\mathcal {G}_5$). Generalized distrust, hopelessness, lack of agency, anomy. The world belongs to others, who have power. People have to accept this situation in order to avoid suffering even more.

**Table 1:** The 22 regions analyzed (NUTS2 territories) divided by country.

Region names and acronyms			
Denmark	Netherlands	United Kingdom	Italy
Denmark	North Netherlands	East Midlands	Center Italy
(DK)	(NL N)	(UK EM)	(IT C)
	East Netherlands	East of England	South Italy
	(NL E)	(UK EE)	(IT S)
	West Netherlands	Greater London	North East Italy
	(NL W)	(UK GL)	(IT NE)
	South Netherlands	North East England	North West Italy
	(NL S)	(UK NE)	(IT NO)
		North West England	Italian Islands
		(UK NW)	(IT I)
		Northern Ireland	
		(UK NI)	
		Scotland	
		(UK S)	
		South East England	
		(UK SE)	
		South West England	
		(UK SW)	
		Wales	
		(UK W)	
		West Midlands	
		(UK WM)	
		Yorkshire and the Humber	
		(UK YH)	

Symbolic universes are more than beliefs—each of them defines a mode of being-in-the-world that shapes the actor’s way of feeling, thinking, and acting—in the final analysis, his/her social identity^28^. Several studies have highlighted the role played by symbolic universes in motivating and channelling social and political behaviour. They proved to orient voting behavior both in Italy^47^ and at the Brexit referendum^48^; again, they proved to be associated with the way relevant topics (immigration, Islam, homosexuality, health, participation and democracy, subjectivity) are represented in newspapers^29^ as well as with the attitude towards vaccination^49^. Moreover, symbolic universes show different levels of trust in institutions, preference for in-group over out-group members, sense of community, perceived quality of the interpersonal bond, attitudes towards foreigners, adhesion to universalist versus self-centered values^28,50^

Taken as a whole, the findings reported above provide convergent support to the conclusion that the map of symbolic universes supplies a reliable way of measuring the three parameters of ECHO-EGN which embed cultural pluralism. For each NUTS2 population, we set the size of the ECHO-EGN groups in accordance to the size of the symbolic universes in that population (see Table 2). Moreover, the levels of Homophily, Endogamic cooperation, and Civicness of each ECHO-EGN cultural group was set in accordance to psycho-social and cultural characteristics of the corresponding symbolic universes, as measured by Salvatore and colleagues^29^ (Endogamic cooperation-cf. table 4.10, p. 156), and Mannarini and colleagues^47^ (Homophily and Civicness: data not reported by the study, available on request)

**Table 2:** Distribution of the Symbolic Universes in each of the sampled 22 regional populations. The reported values are approximated to the second decimal place, and hence some rows may not exactly sum up to 1.

*r*	Acronym	$\delta _{\mathcal {G}_1, r}$	$\delta _{\mathcal {G}_2, r}$	$\delta _{\mathcal {G}_3, r}$	$\delta _{\mathcal {G}_4, r}$	$\delta _{\mathcal {G}_5, r}$
1	DK	0.13	0.34	0.17	0.27	0.09
2	NL N	0.03	0.27	0.27	0.30	0.13
3	NL O	0.09	0.29	0.22	0.30	0.09
4	NL W	0.05	0.28	0.21	0.37	0.10
5	NL S	0.04	0.36	0.16	0.36	0.09
6	UK EM	0.10	0.33	0.05	0.41	0.11
7	UK EE	0.11	0.38	0.06	0.33	0.12
8	UK GL	0.13	0.32	0.06	0.32	0.17
9	UK NEE	0.11	0.34	0.07	0.35	0.13
10	UK NWE	0.10	0.22	0.08	0.49	0.11
11	UK NI	0.16	0.26	0.05	0.37	0.16
12	UK S	0.11	0.17	0.16	0.49	0.06
13	UK SEE	0.13	0.32	0.11	0.25	0.19
14	UK SWE	0.14	0.35	0.08	0.35	0.08
15	UK W	0.05	0.35	0.10	0.39	0.11
16	UK WM	0.04	0.31	0.15	0.34	0.15
17	UK YH	0.16	0.24	0.13	0.32	0.16
18	IT C	0.16	0.26	0.01	0.39	0.18
19	IT S	0.12	0.20	0.00	0.42	0.26
20	IT NE	0.11	0.22	0.02	0.42	0.23
21	IT NO	0.10	0.29	0.02	0.40	0.18
22	IT I	0.12	0.22	0.03	0.36	0.27

More particularly, each group’s Endogamic cooperation was measured in terms of the corresponding symbolic universe’s average level of *Positive Attitude Towards Foreigners* (PATF). This measure is a 4-item subscale of the Prejudice Scale estimating the self-reported propensity to engage with foreigners in social and work contexts^51^. Accordingly, we adopt it as a proxy of the propensity to cooperate with out-group members. For the purpose of the model, we computed the scores in accordance to the following formula: $e_g= \frac{1-PATF_g}{2}$. Thus, the higher the value of $e_g$, the higher the Endogamic cooperation.

Homophily was measured in terms of each symbolic universe’s average level on the *Ethnic scale* (ES). The Ethnic scale is one of the two subscales of the National Identity Scale^52^. It measures the view of identity as based on ethnic and blood linkages, juxtaposed to the universalist view of nationality as based on adhesion to rule of law and citizenship. Accordingly, this index lends itself to be interpreted as a proxy of the preference to relate with the in-group with respect to the out-group (for data supporting this interpretation, see^53^ and^54^). For the purpose of the model, we computed the scores in accordance to the following formula: $h_g= \frac{1+ES_g}{2}$.

Civicness was measured in terms of each symbolic universe’s average level on the *Civic Involvement Scale*(CIS). The Civic Involvement Scale is a measure focused specifically on the estimation of the level of valorization of civic rules. To fit the meaning of the measure, scores were inverted^55^.

Table 3 reports the values of Endogamic cooperation $e_g$, Homophily $h_g$ and Civicness $c_g$ parameters, as well as the the payoff parameters $T^{\mathrm {A}}_g$ and $S^{\mathrm {A}}_g$ for each symbolic universe.

**Table 3:** Parameter setting for each Symbolic Universe. The values $T^{\mathrm {A}}_g$ and $S^{\mathrm {A}}_g$ are evaluated according to equations (2) and (3). The value of $T^{\mathrm {N}}$ is 4, while $S^{\mathrm {N}}= -1$.

**SU name**	$e_g$	$c_g$	$h_g$	$T^{\mathrm {A}}_g$	$S^{\mathrm {A}}_g$
Ordered universe ($\mathcal {G}_1$)	0.22	0.38	0.25	3.11	$-0.78$
Interpersonal bond ($\mathcal {G}_2$)	0.37	0.06	0.48	2.52	$-0.63$
Caring society ($\mathcal {G}_3$)	0.34	0.64	0.34	2.63	$-0.66$
Niche of belongingness ($\mathcal {G}_4$)	0.51	$-0.22$	0.58	1.97	$-0.49$
Others’ world ($\mathcal {G}_5$)	0.71	0.90	0.52	1.17	$-0.29$

Moreover, a further measure of groups was used: *Individual Propensity to Cooperate (IPC)*. It was estimated in terms of each symbolic universe’s average level of *Agreeableness*—a self-report measure of the subject’s propensity to be trustful, open to cooperation (cf.^29^, p. 155). Agreeableness is one sub-scale of the TIPI questionnaire^56^, a short instrument (10 items) used for assessing the Big Five dimensions of personality. IPC was used for validation purposes, rather than for setting the models’ parameters.

Estimation of the symbolic universes’ levels of attitudes towards foreigners and Agreeableness were retrieved from Salvatore and colleagues (^29^, Annex 3, tables 4.3 and 4.10); data concerning the Ethnic Scale and the Civic Involvement Scale were obtained from Mannarini and colleagues^47^, a study performed on an Italian sample.

### Validation dataset

We consider populations of $R=22$ regions, corresponding to 22 NUTS2 territories comprising four European countries—Denmark, Italy, Netherlands, and UK—as reported in Table 1. These populations were selected on the basis of a convenience criterion—populations included are those for which reliable recent information on cultural pluralism were available by Salvatore and colleagues^29^ and Mannarini and colleagues^47^.

### Indexes of population’s cooperation

As proxy of the observable level of cooperation, two *ad hoc* indicators were used: Trust in people and Trust in institutions, according to the European Social Survey (ESS) dataset^57^ (cf. Table 2). Hereafter, these two indicators will be referred to as $Y^{\mathrm {P}}= [Y^{\mathrm {P}}_1, \ldots , Y^{\mathrm {P}}_r, \ldots , Y^{\mathrm {P}}_{R}]$ and $Y^{\mathrm {I}}= [Y^{\mathrm {I}}_1, \ldots , Y^{\mathrm {I}}_r, \ldots , Y^{\mathrm {I}}_{R}]$, where each element stands for one of the *R* regions considered. More specifically, the value $Y^{\mathrm {P}}_{r}$ was obtained as the sum of the 3 items concerning the perception of trustworthiness of people—(a) people can be trusted, (b) people try to be fair, (c) people try to be helpful-, in the *r*-th region, and it ranges in the interval [0, 30], while the value $Y^{\mathrm {I}}_r$ relative to the *r*-th region, is the sum of the 7 EES items concerning the level of trust in regional, national and supranational institutions—(a) country’s Parliament, (b) legal system, (c) police, (d) politicians, (e) political parties, (f) European Parliament, (g) United Nations-, and it ranges in the interval [0, 70]. Data from all populations were obtained by ESS round 8 (2016–2017), with the exception of Denmark, having round 7 (2015–2016) as source (the 2016–2017 round not being available in that case). For both $Y^{\mathrm {P}}$ and $Y^{\mathrm {I}}$ data, also the corresponding standard deviations, $\sigma ^{\mathrm {P}}_r$ and $\sigma ^{\mathrm {I}}_r$ for all the *R* regions, were considered.

All validation data $Y^{\mathrm {P}}$, $Y^{\mathrm {I}}$, $\sigma ^{\mathrm {P}}$, $\sigma ^{\mathrm {I}}$ and $\alpha$ (IPC) are reported in Tables 4 and 5, respectively.

**Table 4:** Validation data: trust in people $Y^{\mathrm {P}}$, trust in institution $Y^{\mathrm {I}}$ and the corresponding standard deviations $\sigma ^{\mathrm {P}}$ and $\sigma ^{\mathrm {I}}$ for each region considered.

**(a)**				
**Region**	$Y^{\mathrm {P}}$	$Y^{\mathrm {I}}$	$\sigma ^{\mathrm {P}}$	$\sigma ^{\mathrm {I}}$
DK	20.20	42.31	4.44	11.86
NL N	18.83	38.45	4.09	10.32
NL E	18.46	39.23	4.24	10.67
NL W	18.35	39.47	4.21	11.03
NL S	17.70	38.05	4.42	11.03
UK EM	16.99	35.22	4.41	12.39
UK EE	16.79	33.42	4.49	11.92
UK GL	16.84	35.59	5.28	12.06
UK NE	16.97	33.43	5.19	13.12
UK NW	16.20	32.01	4.96	14.01
UK NI	15.99	32.20	6.16	14.63
UK SC	18.50	33.47	4.90	11.17
UK SE	17.09	35.74	4.93	10.98
UK SW	18.30	33.64	4.52	11.87
UK W	16.44	32.50	5.17	12.68
UK WM	15.92	30.45	5.60	13.33
UK YH	16.25	31.99	4.83	13.69
IT C	14.08	27.00	6.04	14.81
IT S	12.83	24.49	5.93	13.74
IT NE	13.66	28.27	6.07	13.04
IT NO	14.06	28.69	5.72	13.16
IT I	12.91	24.63	5.88	12.99

**Table 5:** Validation data: IPC (agreeableness) data for each symbolic universe.

**Group**	**IPC** $\alpha _g$
$\mathcal {G}_1$	10.00
$\mathcal {G}_2$	9.77
$\mathcal {G}_3$	10.28
$\mathcal {G}_4$	9.48
$\mathcal {G}_5$	9.75

### Procedure

Using the above setup, for each region, the ECHO-EGN model (5) was simulated until a steady state was reached. For each simulation, cultural parameters were set in accordance to the values reported in Table 3. More particularly, for each region, several numerical experiments were performed by developing 100 numerical solutions of the model. For each solution, the initial condition provided was randomly generated with uniform distribution in the set $(0, 1)^N$. Analogously, in each simulation the random networks were generated according to the procedure described in Appendix A.

### Control experiment

The control experiment was developed by assuming a model of social network approximating the unitary agent assumption. To this aim, Endogamic cooperation and Civicness parameters were set equal to the average evaluated over the SUs, thus assuming that all players have the same behavior, independently of the group they belong to. Practically, the Endogamic cooperation and Civicness parameters were set equal to the average of the values reported in Table 3 for all SUs.

Finally, in order to enable the control simulation to provide between-regions variability, the Homophily parameter was kept in the control model too. Thus, also in the control model the group differentiation was maintained, but only for setting up the Homophily. In so doing, the control condition enabled a specific estimate to be made of the difference of simulation performance due to two cultural parameters (Endogamic cooperation and Civicness). Moreover, with this design, the control model was able to check the alternative hypothesis that the efficacy of the ECHO-EGN simulation was due to the size of the groups.

For each region, the control outputs were obtained by means of the same procedure used for the ECHO-EGN simulations, i.e. 100 experiments, with randomly generated initial condition.

### Results

The average cooperation $X_\mathcal {V}$ and its standard deviation $\sigma ^{\mathrm {X}_\mathcal {V}}$ obtained for each of the 100 numerical solutions of the ECHO-EGN model were compared with real dataset $Y^{\mathrm {P}}$, $Y^{\mathrm {I}}$, $\sigma ^{\mathrm {P}}$ and $\sigma ^{\mathrm {I}}$ by means of the Pearson correlation coefficient *r*. Moreover, the Pearson correlation coefficient was computed as a measure of the association between the level of cooperation (i.e. the values $\alpha$ of IPC, reported in Table 5) of each natural group/symbolic universe over the whole $R=22$ sample, and the corresponding level generated by the simulation models.

**Figure 2:**
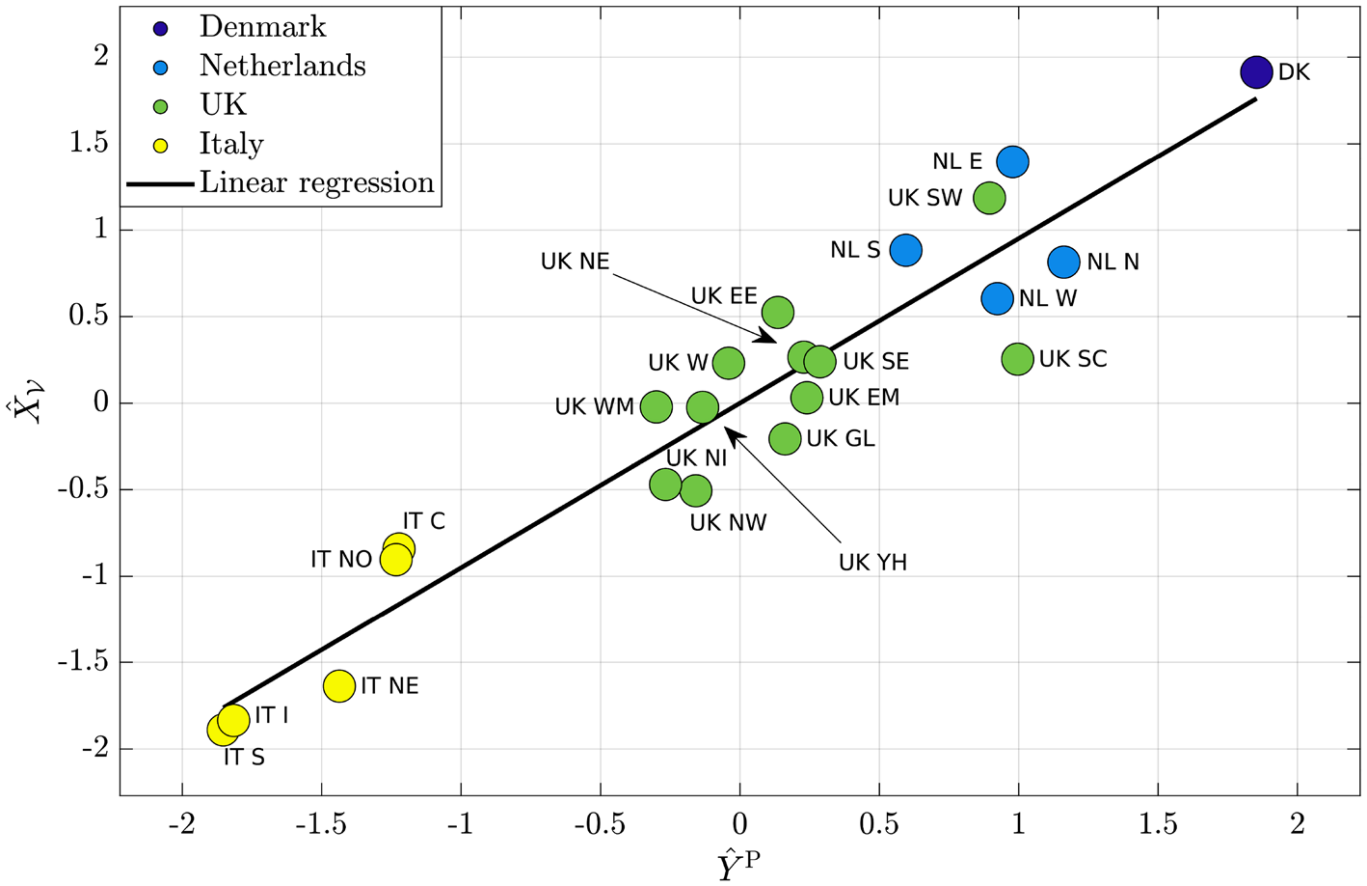
Comparison of standardized dataset $\hat{Y}^{\mathrm {P}}$ with standardized simulation $\hat{X}_{\mathcal {V}}$.

Figure 2 reports, for each region, the corresponding $Y^{\mathrm {P}}$ value and the asymptotic simulated cooperation averaged over the whole population. In particular, we report their standardized version, $\hat{Y}^{\mathrm {P}}$ on *x*-axis and $\hat{X}_{\mathcal {V}}$ on *y*-axis, where $\hat{z} = \frac{z-\langle z \rangle }{\sigma ^z}$. Different colors have been used to distinguish among countries: dark blue for Denmark, light blue for Netherlands, green for UK and yellow for Italy. The black line represents the linear regression, and its closeness to data reveal the very strong correlation between real data and model simulations. Indeed, the slope of the regression line, corresponding to the correlation coefficient, is 0.95, while the offset is almost 0. The *p*-value, indicating the statistical significance of the correlation coefficient, is $1.3 \cdot 10^{-11}$, while $R^2$, representing the quality of the regression line, is 0.99. It is interesting to observe the presence of clusters, clearly identifying each country considered on both real data and model simulations. Denmark and Netherlands exhibit the highest levels of cooperation, UK shows intermediate levels, while the lowest levels is observed for Italy.

**Figure 3:**
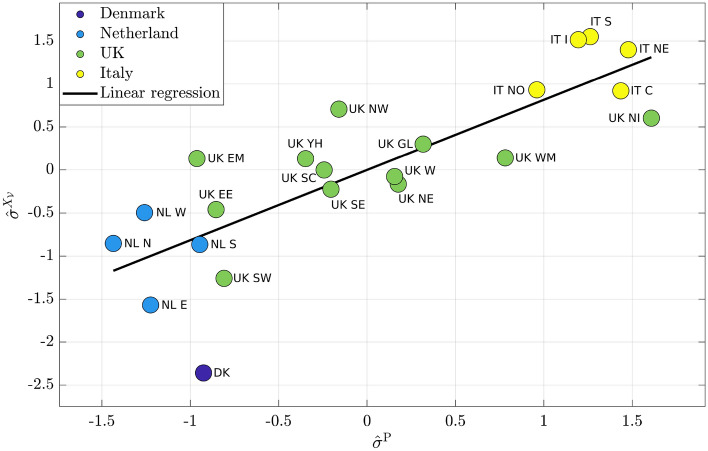
Comparison of standardized dataset $\hat{\sigma }^{\mathrm {P}}$ with standardized simulation $\hat{\sigma }^{X_\mathcal {V}}$.

In Fig. 3, standardized $\sigma ^{\mathrm {P}}$, ($\hat{\sigma }^{\mathrm {P}}$ on *x*-axis) and standardized $\sigma ^{\mathrm {X}_\mathcal {V}}$ ($\hat{\sigma }^{\mathrm {X}_\mathcal {V}}$ on *y*-axis) are reported for all regions. The slope of the regression line is 0.82, while the offset is almost 0. The *p*-value is $3.8 \cdot 10^{-6}$, while $R^2 = 0.97$. Country clusters are present here too; in this case the highest levels of the standard deviation are shown by the measures referring to Italy, denoting a higher heterogeneity of the $Y^{\mathrm {P}}$ data and of simulated cooperation. UK presents intermediate values, while Denmark and Netherlands prove to have the smallest ones.

**Figure 4:**
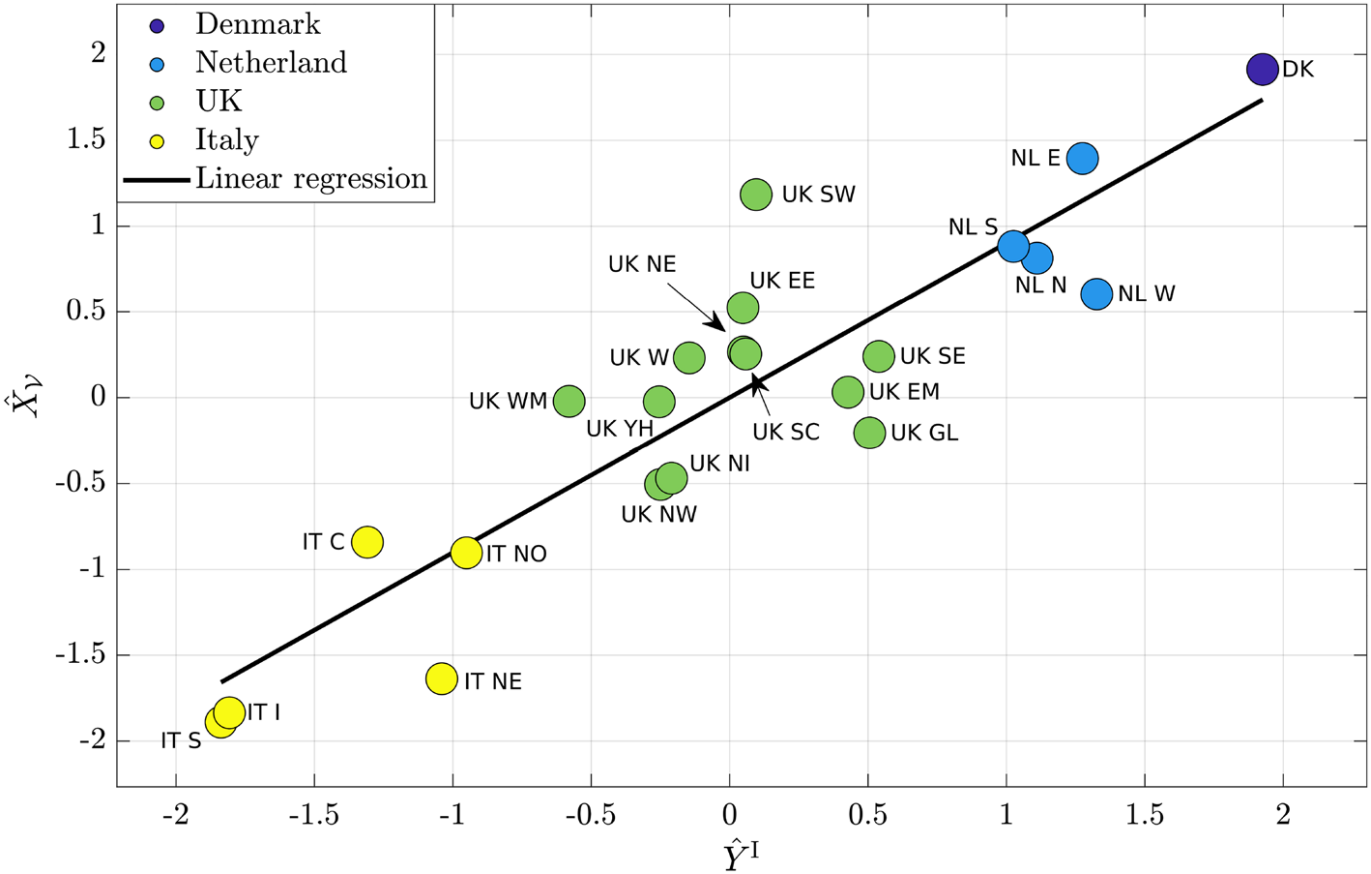
Comparison of standardized dataset $\hat{\sigma }^{\mathrm {I}}$ with standardized simulation $\hat{\sigma }^{X_\mathcal {V}}$.

**Figure 5:**
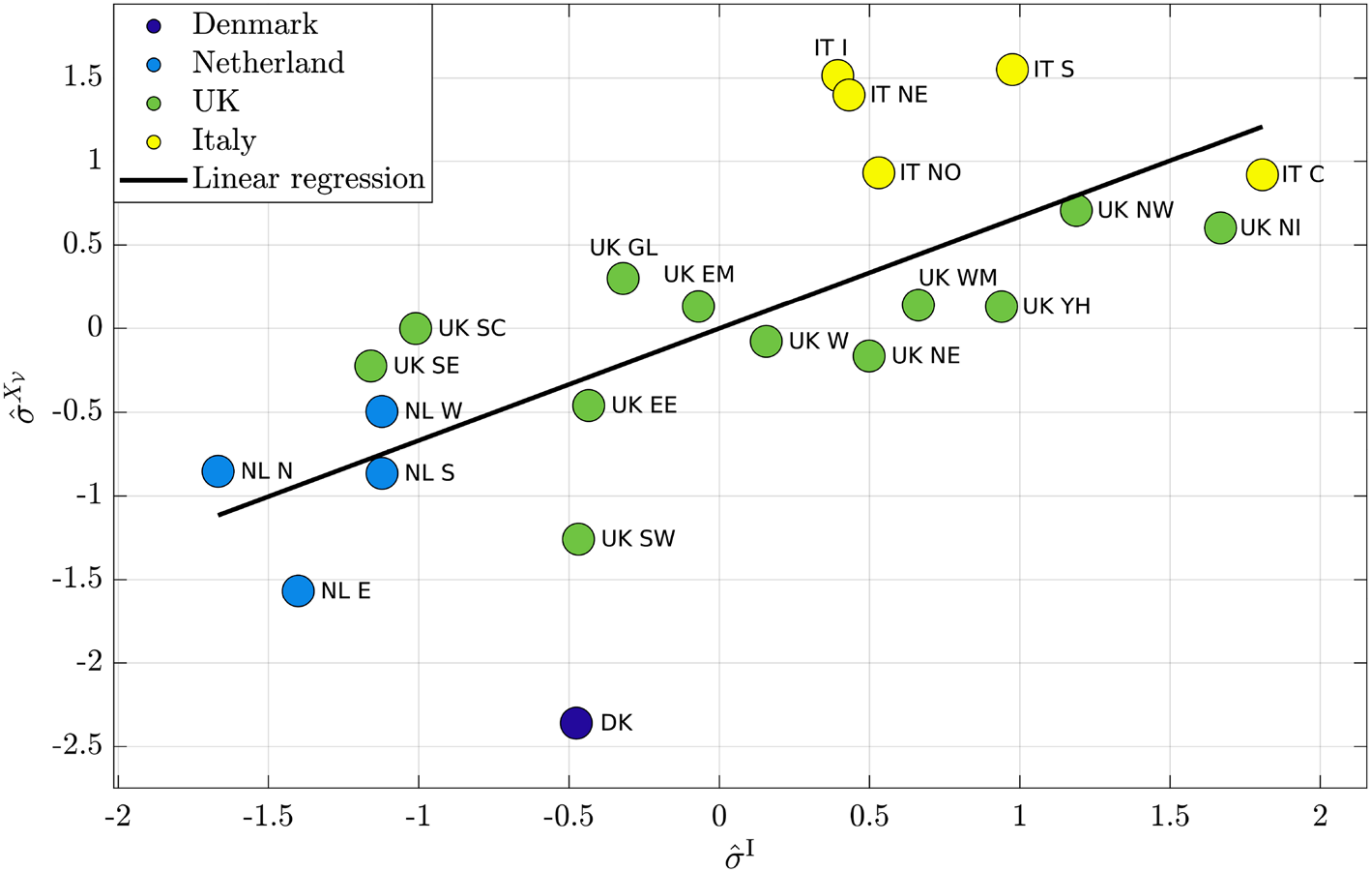
Comparison of standardized dataset $\hat{\sigma }^{\mathrm {I}}$ with standardized simulation $\hat{\sigma }^{X_\mathcal {V}}$.

Similar results were obtained by analyzing $Y^{\mathrm {I}}$ data. Figure 4 reports, for each region, the standardized $Y^{\mathrm {I}}$ value ($\hat{Y}^{\mathrm {I}}$ on *x*-axis) and the standardized $X_\mathcal {V}$ ($\hat{X}_{\mathcal {V}}$ on *y*-axis). The slope of the regression line is 0.9, while the offset is almost 0. The *p*-value is $9.8 \cdot 10^{-9}$, while $R^2 = 0.99$. The presence of country clusters is observed and again, Denmark and Netherlands exhibit the highest levels of $Y^{\mathrm {I}}$ data and the average simulated cooperation, UK shows intermediate levels, while the lowest results are observed in Italy.

In Fig. 5, the standardized value of $\sigma ^{\mathrm {I}}$ ($\hat{\sigma }^{\mathrm {I}}$ on *x*-axis) and standardized $\sigma ^{\mathrm {X}_\mathcal {V}}$ ($\hat{\sigma }^{\mathrm {X}_\mathcal {V}}$ on *y*-axis) are reported for each region. The slope of the regression line is 0.67, while the offset is almost 0. The *p*-value is $6.6 \cdot 10^{-4}$, while $R^2 = 0.89$. Also in this case, highest levels of the standard deviation are reached by Italy, denoting higher heterogeneity of data and simulations. UK shows intermediate values, while Denmark and Netherlands prove to have the lowest ones.

**Figure 6:**
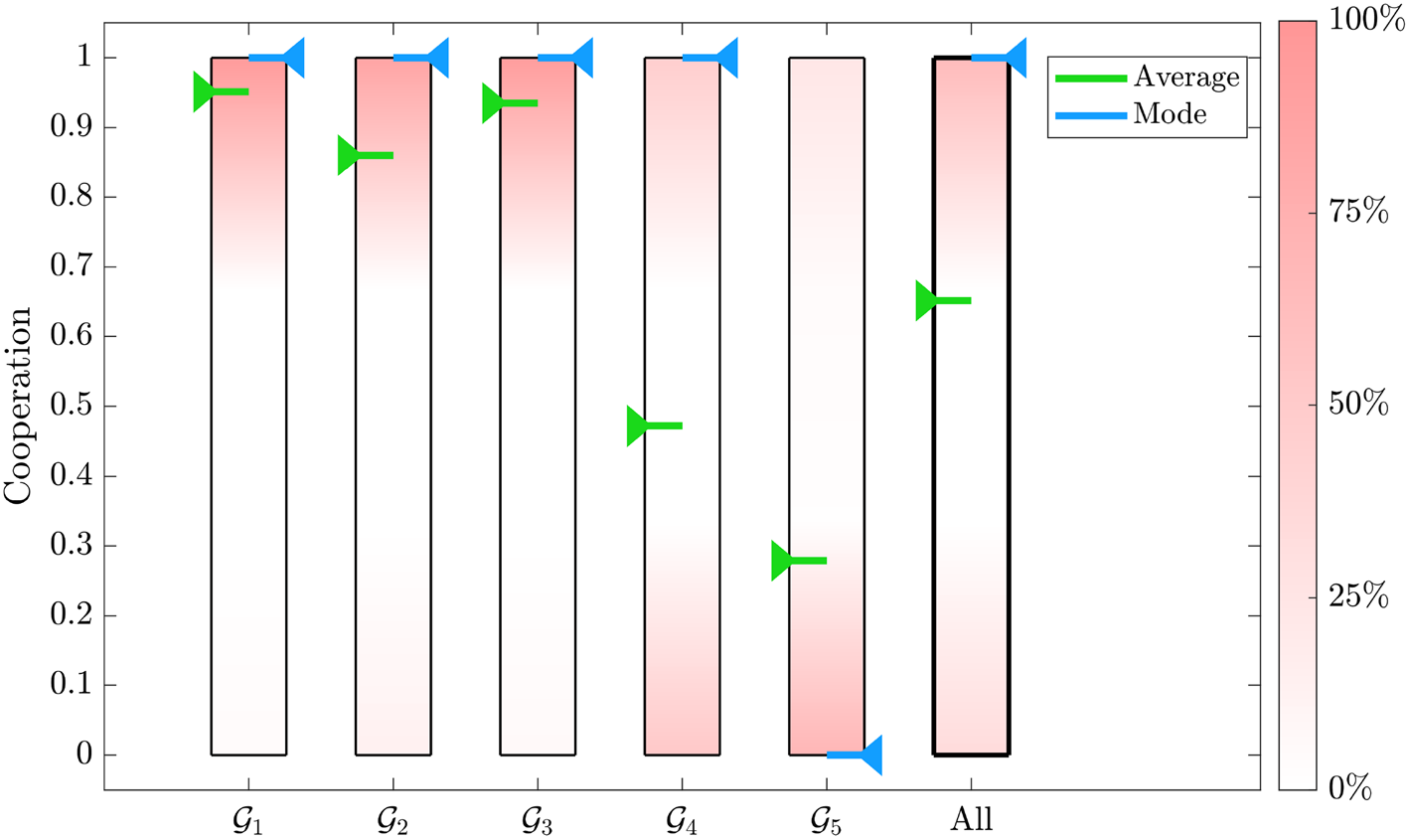
Distribution of the cooperation level of each group and of the whole population. Data refer to the asymptotic cooperation of players belonging to all 22 regions, obtained by the 100 trials.

The distribution of the cooperation level of each group and of the whole population observed in these experiments are reported in Fig. 6, where high concentrations of individuals with a given cooperation level are represented by pink shading. Green and blue arrows denote the average and the mode calculated for each group and over the whole population, respectively.

**Figure 7:**
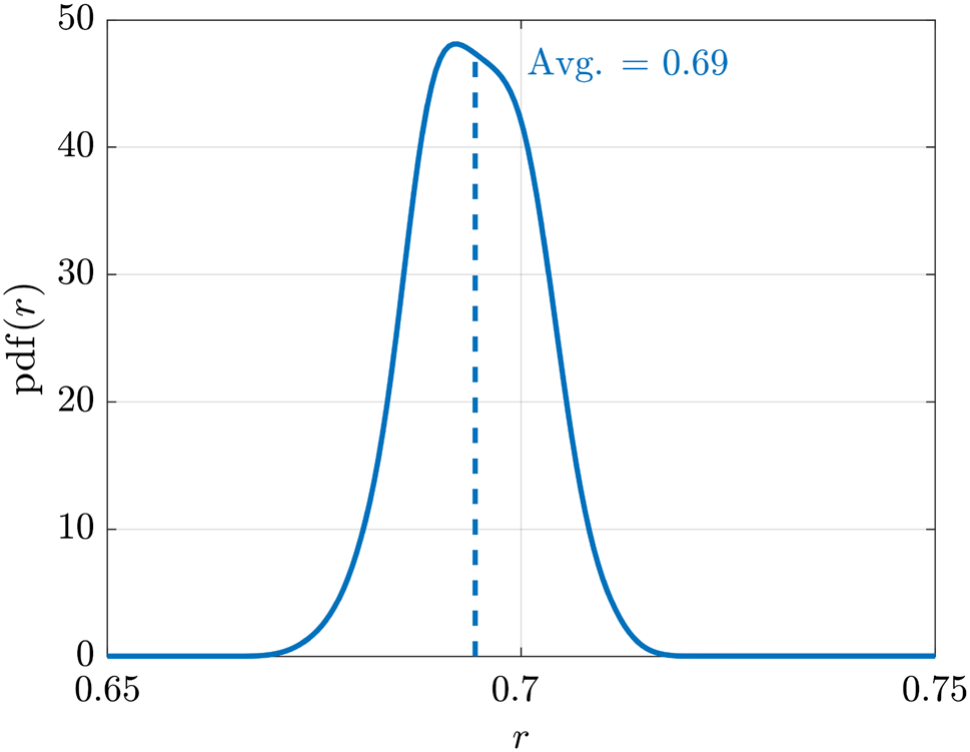
Probability density functions over 100 experiments of the correlations between the average group cooperation $X_{\mathcal {G}}$ and the IPC dataset $\alpha$ centered on the average correlation.

An additional validation was carried out by comparing the average cooperation level recorded for each group of each simulation model and the average Individual Propensity to Cooperate (the variable $\alpha$ of Table 5) of each corresponding natural group. The corresponding estimated probability density function of the correlation $r(X_\mathcal {G}, \alpha )$ is depicted in Fig. 7. The average correlation is $r = 0.69$.

**Figure 8:**
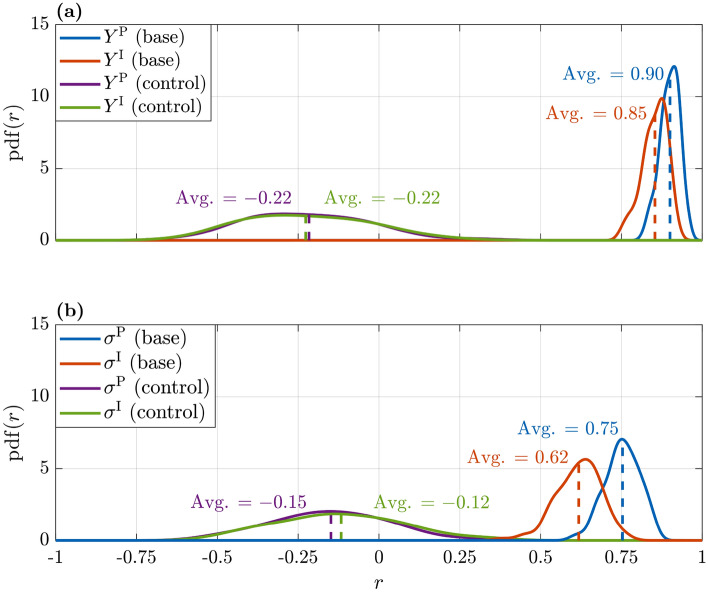
Probability density functions over 100 experiments. Subplot (a): correlations of model simulations and $Y^{\mathrm {P}}$ and $Y^{\mathrm {I}}$ datasets ($r(X_\mathcal {V}, Y^{\mathrm {P}})$ in blue and $r(X_\mathcal {V}, Y^{\mathrm {I}})$ in red). The probability density functions obtained with the control experiments are reported in purple and in green. In this case, $e_g = 0.43 ~\forall g$ and $c_g = 0.13 ~\forall g$. Subplot (b): correlations model simulations and the standard deviation of $\sigma ^{\mathrm {P}}$ and $\sigma ^{\mathrm {I}}$ datasets x (blue and red for the base experiment, purple and green for the control one). Dashed vertical lines are centered on the average correlations.

In order to validate the choice of the parameters, we performed a control experiment as described in section “Control experiment”. In this way, we assumed that all players behave in the same way with respect to the external individual (i.e. constant Endogamic cooperation), and with respect to their attitude to self-regulation of defection (i.e. constant Civicness). Figure 8.a reports the estimated probability density function (pdf) of the correlation coefficients $r(X_\mathcal {V}, Y^{\mathrm {P}})$ (blue for the base experiment, purple for the control one) and $r(X_\mathcal {V}, Y^{\mathrm {I}})$ (red for the base experiment, green for the control one), while Fig. 8.b shows the estimated pdf of the correlation coefficients $r(\sigma ^{\mathrm {X}_\mathcal {V}}, \sigma ^{\mathrm {P}})$ (blue for the base experiment, purple for the control one) and $r(\sigma ^{\mathrm {X}_\mathcal {V}}, \sigma ^{\mathrm {I}})$ (red for the base experiment, green for the control one). The average correlation values correspond to the dashed vertical lines. The highest correlations are observed for both the dataset $Y^{\mathrm {P}}$ ($r=0.93$) and $\sigma ^{\mathrm {P}}$ ($r=0.75$) in the base experiment (blue), while the control experiment (purple) produces uncorrelated results ($r=-0.22$ for $Y^{\mathrm {P}}$ and $r=-0.15$ for $\sigma ^{\mathrm {P}}$). High significant correlations are also found for the $Y^{\mathrm {I}}$ ($r=0.85$) and $\sigma ^{\mathrm {I}}$ ($r=0.65$) datasets, while in the control experiment we observe low values ($r=-0.22$ for $Y^{\mathrm {I}}$ and $r=-0.15$ for $\sigma ^{\mathrm {I}}$). It is clear that in all cases investigated, the performances of the control experiment are much lower than those of the base model, where the different values of Endogamic cooperation and of Civicness are considered. One can also observe that the dispersion of the distributions reported in panel (a) are lower than the ones reported in panel (b).

### Discussion

The output of the simulation study provided convergent support to the ecological validity of ECHO-EGN. The very high correlations between the level of cooperation of the natural groups and the corresponding ECHO-EGN models showed that the latter is able to simulate natural social networks quite efficaciously. The ECHO-EGN’s simulation capacity resulted almost absolute when the trust in people index is used ($r=0.93$); it is however very high ($r=0.85$) in the case of the trust in institution index. This difference may be due to the fact that in the latter case the index involves institutions that are transversal to the territorial populations (e.g. European Union and United Nations) and therefore could be less reflective of the specificity of each local population. The high correlation between the natural and simulated within-population variability of the cooperation ($r=0.75$ and $r=0.65$, respectively for trust in people and trust in institutions) provides further support to the ECHO-EGN ecological validity—this result highlights that the ECHO-EGN is not only able to simulate the global cooperation level of the natural social networks, but also, more importantly, its distribution within the population.

Again, it is worth noticing that the ECHO-EGN model proved able to simulate quite efficaciously the cooperation of the five segments of natural social groups defined by the symbolic universes ($r=0.69$) too. This is a further cross-validation of the ECHO-EGN, provided by an indirect source—namely an independent measure (Individual Propensity to Cooperate) which was not involved in the model set-up.

This convergent evidence is still more significant if one considers that no fine tuning of the model parameters was applied; indeed, parameters were set up by means of a priori knowledge only—i.e. the data on the size and psycho-social characteristics of the cultural segments analyzed by Salvatore and colleagues^29^ and Mannarini and colleagues^47^. In this the findings of the validation test lead to conclude that the ECHO-EGN embeds significantly the psycho-social processes, which drive the emergence of cooperation in social networks.

Finally, the fact that the control model—i.e. the model which embeds two out of three homogeneous cultural parameters—proves to be unable to simulate natural social networks (correlation between control simulations and actual populations is close to 0 or negative in all comparisons) supports the conclusion that the ECHO-EGN ecological validity is due to the parameters mapping the cultural variability of population.

In sum, the main experiment presents significantly higher correlations, thus showing that the introduction of the symbolic universes and the mechanisms of Endogamic cooperation, Civicness and Homophily in the mathematical model accounts reasonably well for traits which regulate the cooperative behavior in social networks.

## Analytical results

### Constraints on cooperation

In this section we report some theoretical results concerning the stability of steady states of the ECHO-EGN equation (5), i.e. constant solutions to which the system dynamics will eventually converge. These states can be found by assuming that $\dot{\mathbf{x }} = 0$, and they embed peculiar properties of asymptotic dynamics of individuals and population as well. In our study, we consider the steady states $\mathbf{x }^{ALLC}$ and $\mathbf{x }^{ALLD}$ of system (5), which represent the situations where all players assume a fully cooperative or fully defective asymptotic behavior, respectively.

#### Theorem 1

*If*$$\displaystyle \beta _v > \eta _v ~\forall v \in \mathcal {V}$$, *where*

$$\begin{aligned} \eta _v = k^{\mathrm {A}}_v + k^{\mathrm {N}}_v \displaystyle \frac{1-T^{\mathrm {N}}}{1-T^{\mathrm {A}}_g}, \end{aligned}$$

*then the steady state*$\mathbf{x }^\text {ALLC}$*is asymptotically stable.*

#### Remark 1

$T^{\mathrm {N}}> T^{\mathrm {A}}_g$ if and only if $\eta _v > k_v$.

The Theorem essentially states that Endogamic cooperation restrains cooperation. Indeed, in a social network without Endogamic cooperation, the asymptotic stability of $\mathbf{x}^{ALLC}$ requires that the self-regulation parameters $\beta _v$ exceed the degree of the player (see Theorem 3 in^41^). On the other hand, since $\eta _v > k_v$ in accordance to Remark 1, to guarantee the asymptotic stability of the fully cooperative steady state $\mathbf{x }^{ALLC}$ in presence of endogamic groups, the members of these groups must have stronger self-regulation parameters, although they show a reduced temptation to defect when playing with the members of the same group ($T^{\mathrm {A}}_g < T^{\mathrm {N}}$).

#### Theorem 2

*If*$$\beta _v < \zeta _v ~\forall v \in \mathcal {V}$$, *where*

$$\begin{aligned} \zeta _v = \displaystyle k^{\mathrm {A}}_v + k^{\mathrm {N}}_v \displaystyle \frac{S^{\mathrm {N}}}{S^{\mathrm {A}}_g}, \end{aligned}$$

*then the steady state*$\mathbf{x }^\text {ALLD}$*is asymptotically stable.*

#### Remark 2

$|S^{\mathrm {N}}| > |S^{\mathrm {A}}_g|$ if and only if $\zeta _v > k_v$.

The Theorem essentially states that Endogamic cooperation fosters defection. Indeed, in a social network without Endogamic cooperation, the asymptotic stability of $\mathbf{x}^{ALLD}$ requires that the self-regulation parameters $\beta _v$ is below the degree of the player (see Theorem 4 in^41^). On the other hand, since $\zeta _v > k_v$ in accordance to Remark 2, despite the fact that affine players have less fear of being betrayed by individuals belonging to the same group than all other individuals ($|S^{\mathrm {A}}_g| < |S^{\mathrm {N}}|$), their behavior is still defective for larger values of the self-regulation parameter than when all players use the same payoff matrix $\mathbf{B}^{\mathrm {N}}$.

The proofs of Theorems 1 and 2 are reported in Appendix C.

### Theorem interpretation

It is interesting to investigate the relationship between values of parameters $\beta _v$ and the thresholds found in Theorems 1 and 2.

**Table 6:** Relationship between average self-regulation parameters $\beta _v$ and the thresholds of Theorems 1 and 2. Col. 2: the value $\beta _v$ averaged over a given group, over the $R=22$ regions and over the 100 trials. Col. 3: the value of threshold $\eta _v$ averaged over a given group, over the $R=22$ regions and over the 100 trials. Col. 4: Theorem 1 satisfaction (in average). Col. 5: the value the value $\zeta _v$ averaged over a given group, over the $R=22$ regions and over the 100 trials. $\eta _v$ averaged over a given group, over the $R=22$ regions and over the 100 trials. Col. 6: Theorem 2 satisfaction (on average).

Name	Avg. $\beta _v$	Avg. $\eta _v$	Th. (1)	Avg. $\zeta _v$	Th. (2)
$\beta _{\mathcal {G}_1}$	16.1	5.5	Yes	5.1	No
$\beta _{\mathcal {G}_2}$	9.6	6.2	Yes	5.4	No
$\beta _{\mathcal {G}_3}$	15.6	6.5	Yes	5.6	No
$\beta _{\mathcal {G}_4}$	5.3	7.8	No	6.0	Yes
$\beta _{\mathcal {G}_5}$	10.6	41.4	No	9.8	No

To this end, in Table 6 the self-regulation parameters $\beta _v$, averaged over the regions and the simulation trials, are reported in column 2 for each group. Moreover, these values are compared to the same averages of threshold $\eta _v$, found in Theorem 1 (column 3). Additionally, the Theorem 1 satisfaction is indicated in column 4 for each group. We observe that, on the average, all groups except for $\mathcal {G}_4$ and $\mathcal {G}_5$ satisfy Theorem 1. Columns 5 and 6 report similar results on full defection, related to Theorem 2. Notice that in this case, “yes” means that the value of column 2 is lower than the value in column 5, and “no” the opposite, as shown in column 6. Only group $\mathcal {G}_4$ satisfies the requirements of Theorem 2. This means that for this group it is not only more difficult to cooperate, but it is also more easier to defect.

### Discussion

The formal analysis of ECHO-EGN leads to a rather counter-intuitive conclusion. The fact that, as Theorem 1 states, Endogamic cooperation reduces the global level of cooperation, means that the in-group identity and therefore in-group solidarity promotes cooperation locally (i.e. among in-group members) but prevents it globally (i.e. at the level of the whole society). On the other hand, Theorem 2 ensures that when Endogamic cooperation is active, the behavior of individuals can be defective even in the case of high self-regulation. These results have a relevant theoretical implication. Indeed, it is consistent with those that criticize the view of in-group bonds (e.g. conceptualized either as sense of community^58^ or bonding social capital^26^) as the lever to increase cooperation and trust in society. In opposition to this rather popular view (e.g.^59,60^), some authors underline that—when not integrated by universtalist and civic attitudes and values—the involvement in the community/in-group bonds can foster identity motives and closure towards the out-group - therefore, paradoxically, to a global reduction of social cooperation and cohesion^61,62^. Theorems 1 and 2 provide analytical support to this criticism, by showing the role played by the relation with not-affine members and Civicness/self-regulation as strategic resources for cooperation.

## Conclusion

This paper focused on ECHO-EGN—a model of the evolution of cooperation in social networks designed to go beyond the unitary agent assumption, which greatly weakens the realism of the analysis, reducing the ecological validity of theoretical conclusions and related pragmatic implications.

ECHO-EGN models the variability of agents in terms of three major parameters, each of them mapping a cultural component of the inherent pluralism of natural social networks: *Homophily*—i.e. preference to relate with in-group members; *Endogamic cooperation*—i.e. the higher propensity to cooperate with in-group members; the *Civicness*—i.e. the propensity to cooperate in the public domain. These components have been conceived as cultural because they are fostered by cultural norms and related psycho-social drivers.

The validation test on ECHO-EGN shows that, thanks to these cultural parameters, the model reaches capacity to simulate the level of cooperation of natural social networks—from almost full to very high, accordingly to the index of cooperation adopted. Moreover, ECHO-EGN proves to be able to simulate efficaciously the within-population distribution of cooperation as well as the average level of the cultural segments’ propensity to cooperate.

The high performance on the simulation test leads to two complementary conclusions. On the one hand, it supports the validity of the cultural parameters used by ECHO-EGN, legitimizing them as an effective way to map the cultural and psycho-social processes underpinning the dynamics of cooperation. On the other hand, it provides further evidence of the theoretical and methodological soundness of the notion of symbolic universes^28,50^. Indeed, this notion underlies the data used to set up the ECHO-EGN models simulating natural social networks. Therefore, the success of the simulation is an indirect proof of the fact that the conceptual and methodological concept of symbolic universe provides a valid and reliable approach to the cultural analysis of a social group.

The formal analysis of ECHO-EGN provides further food for thought. It shows that cooperation is prevented, rather than fostered, by in-group identity and solidarity. This result is theoretically and practically relevant—it integrates the idea of community as the fundamental resource for promoting social cooperation and development. What the analysis specifically suggests is that valorized community bonds have to be integrated by the restoration of forms of universalism in order to make societies more cohesive and inclusive^34^.

In general, the realism demonstrated by ECHO-EGN has important implications. At the methodological level, it enables a forecast approach, aimed at mapping the impact of the variation of cultural factors on cooperation. Such an approach could have a practical value too—it paves the way for the use of simulation and formal analysis in the design of policies for social cohesion and cooperation. According to this view, ECHO-EGN can be to estimate and/or to model the impact of cultural factors on the natural social networks’ level of cooperation, for the sake of identifying critical drivers and/or setting strategic objectives and/or estimating the consequences of interventions.

Before concluding, it is worth highlighting some limitations of the study. First, the simulation test was based on a rather small sample of units of analysis, collected on a convenience criterion. Thus, even if the sample proved to incorporate relevant geographical variability (it comprises territories from a Mediterranean country and Northern-European countries as well), further analyses, based on more comprehensive samples of population are required to support the generalization of the current conclusion. Second, the set-up of some of the cultural parameters (more particularly, Homophily and Endogamic cooperation) were based on indirect indicators, chosen on a criterion of convenience (i.e the availability of data). Thus, further studies are required to test different, more direct estimations of Homophily, Endogamic cooperation. On the other hand, it has to be noted that if the current estimation of these two parameters should be proved to be imprecise, this would mean that ECHO-EGN would have a further chance to empower its ecological validity. Third, the simulation test used only two criteria (trust in people and trust in institutions) to estimate the convergence between simulated and natural social networks. Further studies will be aimed at analyzing the ECHO-EGN’s ecological validity through other indicators of cooperation—this will be done both to corroborate its realism and to identify the specific aspects of cooperation the model is sensitive to. Finally, we are aware that other components of cultural pluralism need to be taken into account, in order to enhance the ECHO-EGN ecological validity—e.g. the heterogeneous propensity to socialize, further sources of the inherent differences in the agents’ propensity to cooperate, the different temporality agents adopt as the frame of their decisions. The current findings of our work encourage us to see these elements as sources of potential developments of ECHO-EGN, with the prospect of building a mathematical model of the cultural dynamics of cooperation.

## Appendix A: Network generation for grouped populations

Given the region *r* in the set of $R=22$ populations considered in this study, we generated $M=5$ scale-free networks, each of size $N_g = \delta _{r,g} N$ according to Table 2. The scale-free structure is obtained by employing preferential attachment methods. In a nutshell, given the desired average degree $\overline{k}_g$, an initial fully connected network of $m = \frac{\overline{k}_g}{2}$ is generated. Then, further nodes are added to the network, by connecting it via *m* links to the already existing nodes. The probability that a new node is connected to one of the existing ones is proportional to the current degree of those nodes. This procedure is repeated until the number of nodes in the networks is equal to $N_g$.

The networks obtained are not cross connected. In order to create inter-group links and to embed the Homophily property, a rewiring process is implemented. Specifically, for each couple of connected players *v* and *w*, where *v* belongs to group *g*, the link is removed with probability $1-h_g$, and replaced by a new link between *v* and a random player of a group different from *g*. Since the Homophily $h_g$ denotes the probability of one player being connected to an affine one, then the probability of changing an affine link with a non affine one is $1-h_g$. It can therefore be seen that, due to the stochasticity of this process, $k^{\mathrm {A}}_v = h_gk_v$ and $k^{\mathrm {N}}_v = (1-h_g)k_v$ on the average.

Due to the stochasticity of the network, the effective average degree $\overline{k}'_g$ of a group *g* is in general slightly different than the average $\overline{k}_g$. To distinguish the two cases, we introduce the effective parameters $\overline{k}'_g$ and $\overline{k}'$ as $\overline{k}'_g = \frac{1}{N_g}\sum _{v \in \mathcal {G}_g} k_v,$ and $\overline{k}' = \frac{1}{N} \sum _{v \in \mathcal {V}} k_v.$

According to the assumption discussed in section “Homophily”, for which the topology of the connection network is random with a scale-free distribution, we notice that the network obtained by the above procedure is still scale-free. Indeed:

$$\begin{aligned} P(k_v = k)&= \sum _{g=1}^M P(k_v = k | v \in \mathcal {G}_{g})P(v \in \mathcal {G}_{g}) \\&= \sum _{g=1}^M P(k_v = k | v \in \mathcal {G}_{g})\delta _{g} \\&= \sum _{g=1}^M \delta _{g} \frac{1}{2}{\overline{k}_{g}}^2 k^{-3}dk \\&= \frac{1}{2} \left( \sum _{g=1}^M \delta _{g}{\overline{k}_{g}}^2\right) k^{-3}dk \\&= \frac{1}{2} \overline{k}^2 k^{-3}dk, \end{aligned}$$

where $\delta _g$ is the share of group *g* in the considered population and $\overline{k} = \sqrt{\displaystyle \sum _{g=1}^M \delta _{g}{\overline{k}_{g}}^2}$ is the average degree of the resulting complete network.

Since in this paper we assume that $\overline{k}_g = 4$, then $\overline{k} = 4\sqrt{\sum _{g=1}^M \delta _{g}} = 4$.

## Appendix B: Derivation of the ECHO-EGN model

Let’s consider a generic player *v* belonging to group *g*, and another player *w* connected to *v*. We have that:

$$\begin{aligned} T_{v,w} = {\left\{ \begin{array}{ll} T^{\mathrm {N}}&{} \text {if}~w \not \in \mathcal {G}_g \\ T^{\mathrm {A}}_g &{} \text {if}~w \in \mathcal {G}_g \\ \end{array}\right. } ~\text {and} ~S_{v,w} = {\left\{ \begin{array}{ll} S^{\mathrm {N}}&{} \text {if}~w \not \in \mathcal {G}_g \\ S^{\mathrm {A}}_g &{} \text {if}~w \in \mathcal {G}_g \\ \end{array}\right. }. \end{aligned}$$

Moreover, $T_{v,v} = T^{\mathrm {A}}_g$ and $S_{v,v} = S^{\mathrm {A}}_g$, i.e. each player is affine with itself. Accordingly, equation (1) can be rewritten as follows:

$$\begin{aligned} \begin{array}{rcl} \dot{x}_v &{} = x_v(1-x_v) &{} \Bigg \{\displaystyle \sum _{w \in \mathcal {G}_g} a_{v,w} \left[ (1-T_{v,w}-S_{v,w})x_w + S_{v,w}\right] \\ &{} &{} + \displaystyle \sum _{w \in \mathcal {V} \setminus \mathcal {G}_g} a_{v,w} \left[ (1-T_{v,w}-S_{v,w})x_w + S_{v,w}\right] \\ &{} &{} -\beta _v\left[ (1-T_{v,v}-S_{v,v})x_v + S_{v,v}\right] \Bigg \} \\ &{} = x_v(1-x_v) &{} \Bigg \{\displaystyle \sum _{w \in \mathcal {G}_g} a_{v,w} \left[ (1-T^{\mathrm {A}}_{g}-S^{\mathrm {A}}_{g})x_w + S_{v,w}\right] \\ &{} &{} + \displaystyle \sum _{w \in \mathcal {V} \setminus \mathcal {G}_g} a_{v,w} \left[ (1-T^{\mathrm {N}}-S^{\mathrm {N}})x_w + S_{v,w}\right] \\ &{} &{} -\beta _v\left[ (1-T^{\mathrm {A}}_g-S^{\mathrm {A}}_{g})x_v + S_{v,v}\right] \Bigg \} \\ &{} = x_{v}(1 - x_{v}) &{} \displaystyle \Bigg \{ k^{\mathrm {A}}_v\left[ \left( 1-T^{\mathrm {A}}_{g}-S^{\mathrm {A}}_{g}\right) \overline{x}^{\mathrm {A}}_v + S^{\mathrm {A}}_g\right] \\ &{} &{} + k^{\mathrm {N}}_v\left[ \left( 1-T^{\mathrm {N}}-S^{\mathrm {N}}\right) \overline{x}^{\mathrm {N}}_v + S^{\mathrm {N}}\right] \\ &{} &{} - \beta _v\left[ \left( 1-T^{\mathrm {A}}_{g}-S^{\mathrm {A}}_{g}\right) x_v + S^{\mathrm {A}}_{g}\right] \Bigg \}. \end{array} \end{aligned}$$

The last equality corresponds to the ECHO-EGN model described by Eq. (5).

## Appendix C: Proofs of Theorems 1 and 2

In this appendix the proofs of the theoretical results concerning the stability of the steady states $\mathbf{x }^{ALLC}$ and $\mathbf{x}^{ALLD}$ of the ECHO-EGN Eq. (5), referred to in section “Analytical results”, are reported. To this aim, the equations (5) are linearized locally near $\mathbf{x }^{ALLC}$ and $\mathbf{x }^{ALLD}$ by evaluating the entries of the Jacobian matrix

$$\begin{aligned} J(\mathbf{x }) = \left\{ j_{v,w} \mathbf (x) \right\} = \left\{ \frac{\partial \dot{x}_v}{\partial x_w}{} \mathbf (x) \right\} . \end{aligned}$$

It is useful to rewrite equation (5) as follows:

A.6 $$\begin{aligned} \dot{x}_{v} = x_{v}(1 - x_{v}) \displaystyle \left[ k^{\mathrm {A}}_vf^{\mathrm {A}}_{g}(\overline{x}^{\mathrm {A}}_v)+ k^{\mathrm {N}}_vf^{\mathrm {N}}(\overline{x}^{\mathrm {N}}_v) -\beta _vf^{\mathrm {A}}_{g}(x_v)\right] , \end{aligned}$$

where

$$\begin{aligned} f^{\mathrm {A}}_{g}(z) = \left( 1-T^{\mathrm {A}}_{g}-S^{\mathrm {A}}_{g}\right) z + S^{\mathrm {A}}_{g} \end{aligned}$$

and

$$\begin{aligned} f^{\mathrm {N}}(z) = \left( 1-T^{\mathrm {N}}-S^{\mathrm {N}}\right) z + S^{\mathrm {N}}. \end{aligned}$$

In particular, given a player $v \in \mathcal {G}_g$, the diagonal entries of $J(\mathbf{x })$ are:

A.7 $$\begin{aligned} \begin{array}{rcl} \displaystyle \frac{\partial \dot{x}_v}{\partial x_v} &{} = &{} (1-2x_v)\left[ k^{\mathrm {A}}_vf^{\mathrm {A}}_g(\overline{x}^{\mathrm {A}}_v)+ k^{\mathrm {N}}_vf^{\mathrm {N}}(\overline{x}^{\mathrm {N}}_v)-\beta _vf^{\mathrm {A}}_g(x_v)\right] \\ &{} &{} -\beta _v x_v(1-x_v)(1-T^{\mathrm {A}}_g-S^{\mathrm {A}}_g). \end{array} \end{aligned}$$

On the other hand, the off-diagonal entries of $J(\mathbf{x })$ are:

A.8 $$\begin{aligned} \frac{\partial \dot{x}_v}{\partial x_w} = {\left\{ \begin{array}{ll} x_v(1-x_v)(1-T^{\mathrm {N}}-S^{\mathrm {N}}) &{} \text {if}~w \not \in \mathcal {G}_g\\ x_v(1-x_v)(1-T^{\mathrm {A}}_g-S^{\mathrm {A}}_g) &{} \text {if}~w \in \mathcal {G}_g \end{array}\right. }. \end{aligned}$$

From the theory of nonlinear dynamic systems, the stability of a steady state $\mathbf{x }^*$ depends on the sign of the real part of the eigenvalues of the corresponding Jacobian matrix $J(\mathbf{x }^*)$^43^. For $\mathbf{x }^{ALLC}$ and $\mathbf{x }^{ALLD}$, since $x_v \in \{0, 1\}$, then the off-diagonal entries reported in (A.8) are identically null. Therefore, the Jacobian matrix has a diagonal structure, and hence its eigenvalues coincide with the expressions reported in (A.7), i.e. $\lambda _v = j_{v,v}(\mathbf{x }^*) = \frac{\partial \dot{x}_v}{\partial x_v}(\mathbf{x}^*)$. In the following Theorems, we find the sufficient conditions to have negative $\lambda _v ~\forall v \in \{1, \ldots , N\}$.

**Theorem 1.** If $\displaystyle \beta _v > \eta _v ~\forall v \in \mathcal {V}$, where

$$\begin{aligned} \eta _v = k^{\mathrm {A}}_v + k^{\mathrm {N}}_v \displaystyle \frac{1-T^{\mathrm {N}}}{1-T^{\mathrm {A}}_g}, \end{aligned}$$

then the steady state $\mathbf{x }^\text {ALLC}$ is asymptotically stable.

### Proof

Considering a player $v \in \mathcal {G}_g$, from equation (A.7) we get that:

$$\begin{aligned} \begin{array}{ccl} \lambda _v = j_{v,v}(\mathbf{x }^\text {ALLC}) &{}= &{} -(k^{\mathrm {A}}_v f^{\mathrm {A}}_g(1) + k^{\mathrm {N}}_v f^{\mathrm {N}}(1) - \beta _v f^{\mathrm {A}}_g(1)) \nonumber \\ &{}= &{} -(k^{\mathrm {A}}_v(1-T^{\mathrm {A}}_g) + k^{\mathrm {N}}_v(1-T^{\mathrm {N}})-\beta _v (1-T^{\mathrm {A}}_g)) \nonumber \\ &{} = &{} -(1-T^{\mathrm {A}}_g)\left( k^{\mathrm {A}}_v + k^{\mathrm {N}}_v\displaystyle \frac{(1-T^{\mathrm {N}})}{(1-T^{\mathrm {A}}_g)}-\beta _v\right) \nonumber \\ &{} = &{} -(1-T^{\mathrm {A}}_g)\left( \eta _v-\beta _v\right) . \nonumber \end{array}. \end{aligned}$$

Since $T^{\mathrm {A}}_g > 1$ and $\beta _v > \eta _v$, then $\lambda _v < 0$. This holds for any *v*. Hence, all eigenvalues are negative, and $\mathbf{x }^\text {ALLC}$ is asymptotically stable. $\square$

In accordance to Theorem 3 in^41^, we know that, considering a population which uses only the payoff matrix $\mathbf{B}^{\mathrm {N}}$, then $\mathbf{x }^{\text {ALLC}}$ is asymptotically stable if

$$\begin{aligned} \beta _v > k_v ~\forall v. \end{aligned}$$

Comparing the thresholds obtained in Theorem 1 of this paper and in Theorem 3 of^41^, we notice that:

$$\begin{aligned} \eta _v> k_v \iff T^{\mathrm {N}}> T^{\mathrm {A}}_g. \end{aligned}$$

Indeed:

$$\begin{aligned} \begin{array}{rcll} k^{\mathrm {A}}_v + k^{\mathrm {N}}_v \displaystyle \frac{1-T^{\mathrm {N}}}{1-T^{\mathrm {A}}_g} &{}> &{} k_v &{} \iff \\ k^{\mathrm {N}}_v \displaystyle \frac{1-T^{\mathrm {N}}}{1-T^{\mathrm {A}}_g} &{}> &{} k_v-k^{\mathrm {A}}_v = k^{\mathrm {N}}_v &{} \iff \\ \displaystyle \frac{1-T^{\mathrm {N}}}{1-T^{\mathrm {A}}_g} &{}> &{} 1 &{}\iff \\ T^{\mathrm {N}}&{} > &{} T^{\mathrm {A}}_g. \end{array} \end{aligned}$$

This corresponds to Remark 1.

Despite the fact that players have lower temptation to defect towards members of their own group than towards all other individuals ($T^{\mathrm {A}}_g < T^{\mathrm {N}}$), they must possess a stronger self-regulation parameter $\beta _v$ with respect to the case where all players use always the same payoff matrix $\mathbf{B}^{\mathrm {N}}$ to guarantee the asymptotic stability of the fully cooperative steady state $\mathbf{x }^{ALLC}$.

**Theorem 2.***If*$\beta _v < \zeta _v ~\forall v \in \mathcal {V}$, *where*

$$\begin{aligned} \zeta _v = \displaystyle k^{\mathrm {A}}_v + k^{\mathrm {N}}_v \displaystyle \frac{S^{\mathrm {N}}}{S^{\mathrm {A}}_g}, \end{aligned}$$

*then the steady state*$\mathbf{x }^\text {ALLD}$*is asymptotically stable.*

### Proof

Considering a player $v \in \mathcal {G}_g$, from equation (A.7) we get that:

$$\begin{aligned} \begin{array}{ccl} \lambda _v = j_{v,v}(\mathbf{x }^\text {ALLD}) &{} = &{} k^{\mathrm {A}}_v f^{\mathrm {A}}_g(0) + k^{\mathrm {N}}_v f^{\mathrm {N}}(0) - \beta _v f^{\mathrm {A}}_g(0) \\ &{} = &{} k^{\mathrm {A}}_vS^{\mathrm {A}}_g + k^{\mathrm {N}}_v S^{\mathrm {N}}-\beta _v S^{\mathrm {A}}_g \\ &{} = &{} S^{\mathrm {A}}_g\left( k^{\mathrm {A}}_v + k^{\mathrm {N}}_v\displaystyle \frac{S^{\mathrm {N}}}{S^{\mathrm {A}}_g}-\beta _v\right) \\ &{} = &{} S^{\mathrm {A}}_g\left( \zeta _v-\beta _v\right) . \end{array}. \end{aligned}$$

Since $S^{\mathrm {A}}< 0$ and $\beta _v < \zeta _v$, then $\lambda _v < 0$. This holds for any *v*. Hence, all eigenvalues are negative, and $\mathbf{x }^\text {ALLD}$ is asymptotically stable. $\square$

In accordance to Theorem 4 of^41^, we know that, considering a population which uses only the payoff matrix $\mathbf{B}^{\mathrm {N}}$, then $\mathbf{x }^{\text {ALLD}}$ is asymptotically stable if

$$\begin{aligned} \beta _v < k_v ~\forall v. \end{aligned}$$

Comparing the thresholds obtained in Theorem 2 of this paper and in Theorem 4 of^41^, we notice that:

$$\begin{aligned} \zeta _v> k_v \iff |S^{\mathrm {N}}| > |S^{\mathrm {A}}_g|. \end{aligned}$$

Indeed:

$$\begin{aligned} \begin{array}{rcll} k^{\mathrm {A}}_v + k^{\mathrm {N}}_v \displaystyle \frac{S^{\mathrm {N}}}{S^{\mathrm {A}}_g} &{}> &{} k_v &{}\iff \\ k^{\mathrm {N}}_v \displaystyle \frac{S^{\mathrm {N}}}{S^{\mathrm {A}}_g} &{}> &{} k_v-k^{\mathrm {A}}_v = k^{\mathrm {N}}_v &{} \iff \\ \displaystyle \frac{S^{\mathrm {N}}}{S^{\mathrm {A}}_g} &{}> &{} 1 &{} \iff \\ \displaystyle |S^{\mathrm {N}}| &{} > &{} |S^{\mathrm {A}}_g|. \end{array} \end{aligned}$$

This corresponds to Remark 2. Hence, even if affine players are less afraid of being betrayed by affine players than all other individuals ($|S^{\mathrm {A}}_g| < |S^{\mathrm {N}}|$), their behavior is defective also for larger values of the self-regulation parameter with respect to the case where all players use the same payoff matrix $\mathbf{B}^{\mathrm {N}}$.
